# Tim-3 deteriorates neuroinflammatory and neurocyte apoptosis after subarachnoid hemorrhage through the Nrf2/HMGB1 signaling pathway in rats

**DOI:** 10.18632/aging.103796

**Published:** 2020-11-07

**Authors:** Shenquan Guo, Yuanzhi Li, Boyang Wei, Wenchao Liu, Ran Li, Wenping Cheng, Xin Zhang, Xuying He, Xifeng Li, Chuanzhi Duan

**Affiliations:** 1 The National Key Clinical Specialty, The Engineering Technology Research Center of Education Ministry of China, Guangdong Provincial Key Laboratory on Brain Function Repair and Regeneration, Department of Neurosurgery, Zhujiang Hospital, Southern Medical University, Guangzhou, China; 2 Department of Neurosurgery, Affiliated Hengyang Hospital, Southern Medical University (Hengyang Central Hospital), Hengyang, China

**Keywords:** subarachnoid hemorrhage, early brain injury, neuroinflammation, Tim-3, HMGB1

## Abstract

Inflammation is known to play an important role in early brain injury (EBI) after subarachnoid hemorrhage (SAH). T cell immunoglobulin and mucin domain-3 (Tim-3) has emerged as a critical regulator of adaptive and innate immune responses, and has been identified to play a vital role in certain inflammatory diseases; The present study explored the effect of Tim-3 on inflammatory responses and detailed mechanism in EBI following SAH. We investigated the effects of Tim-3 on SAH models established by endovascular puncture method in Sprague–Dawley rats. The present studies revealed that SAH induced a significant inflammatory response and significantly increased Tim-3 expression. Tim-3-AAV administration aggravated neurocyte apoptosis, brain edema, blood-brain barrier permeability, and neurological dysfunction; significantly inhibited Nrf2 expression; and increased HMGB1 expression and secretion of pro-inflammatory cytokines, such as tumor necrosis factor alpha, interleukin (IL)-1 beta, IL-17, and IL-18. However, Tim-3 siRNA or NK252 administration abolished the pro-inflammatory effects of Tim-3. Our results indicate a function for Tim-3 as a molecular player that links neuroinflammation and brain damage after SAH. We reveal that Tim-3 overexpression deteriorates neuroinflammatory and neurocyte apoptosis after subarachnoid hemorrhage through the Nrf2/HMGB1 signaling pathway in rats.

## INTRODUCTION

Subarachnoid hemorrhage (SAH) is a devastating cerebrovascular disease with high mortality, disability and poor outcome, and is caused mostly by ruptured aneurysms and other cerebrovascular emergencies [1, 2]. Delayed cerebral vasospasm has been considered to be the main cause of poor prognosis in patients after SAH, however, clinical conversions are rare and mostly unsuccessful—to date, only nimodipine has been used clinically [3, 4]. Increasing evidence has shown that early brain injury (EBI), occurring in the first 72 h following SAH, contributes to a poor prognosis [5, 6]. Among the multiple mechanisms involved, inflammation has been shown to play an important role in EBI following SAH [7]. However, the specific mechanism of inflammation in EBI following SAH is not well understood.

High-mobility group box 1 (HMBG1), as an alarmin for inflammation, was increased in the CSF of SAH patients among approximately 3000 proteins identified by proteomic analysis of cerebrospinal fluid (CSF), and demonstrated strong involvement in sterile inflammation after SAH [8, 9]. In addition, HMGB1 elevated immediately after SAH and functions as a damage-associated molecular pattern to mediate innate immune responses [10, 11]. As previously reported, the translocation and secretion of HMGB1 from activated immune cells is a highly-regulated process involving multiple mechanisms [11]. Therefore, it is extremely important to regulate the transposition and secretion of HMGB1. NF-E2-related factor 2 (Nrf2) has been reported to suppress the translocation and secretion of HMBG1 as one of the regulatory element of Hemeoxygenase-1 (HO-1) promoter region [12]. In addition, Nrf2 plays a crucial role in maintaining normal physiological processes in the brain [13–15] and is important in the oxidative stress and inflammatory response in EBI after SAH [16]. Previous studies on Nrf2 mainly focus on oxidative stress, but its inflammatory effect should not be underestimated. The Nrf2/HMGB1 pathway may play an important inflammatory role in EBI following SAH.

T-cell immunoglobulin and mucin domain protein (Tim-3) is an immuno-regulation molecule discovered in 2002, which was originally found to be expressed in activated Th1 cells [17, 18]. Subsequently, Tim-3 was found to be expressed in various immune cell types, including natural killer cells, monocytes, microglia/macrophages, mast cells, and dendritic cells [19–22]. Tim-3 is a vital regulatory factor in both innate and adaptive immunity, and is involved in the production of proinflammatory cytokines, such as tumor necrosis factor-α (TNF-α) and interleukin-1β (IL)-1β, which participate in inflammation [23, 24]. Tim-3 is mainly expressed in microglia in the brain and participates in the inflammatory response of central nervous system (CNS) diseases, such as ischemic stroke, multiple sclerosis, experimental autoimmune encephalomyelitis, and cerebral parasitic diseases [24–28]. Interestingly, Tim-3 seems to have the opposite functions under different pathological conditions, with its functional outcomes depending on the cell type and context, even they are all in the CNS. For example, downregulation of Tim-3 appears to promote the transformation of microglia phenotype from M1 to M2 and alleviates the neuroinflammation after ICH, whereas enhancement of TIM-3 signalling appears to ameliorate Th-1-mediated EAE. However, until now, the effect of Tim-3 on the inflammatory response in EBI following SAH has been unclear.

Here, we systematically investigated the possible mechanism that Tim-3 aggravates neuroinflammatory by regulating the Nrf2/HMGB1 pathway by establishing a SAH model in rats. In this study, we provide evidence that Tim-3 aggravates neuroinflammation and augments HMGB1 secretion *via* targeting the Nrf2/HMGB1 pathway in SAH rats. Collectively, our results suggest that Tim-3 may be an important molecular player in the inflammatory role in EBI following SAH. These insights into the link between neuroinflammation and SAH improve our understanding of the functions of Tim-3 and Nrf2/HMGB1 pathway and may contribute to the development of new therapeutic strategies for SAH.

## RESULTS

No significant differences in physiological indicators (body temperature or bodyweight) were observed among rats of the different experimental groups (data not shown). Moreover, we found that there was no statistical difference in Tim-3 protein abundance among the sham groups at each time point.

### Mortality and SAH grade in rats

**Figure 1:**
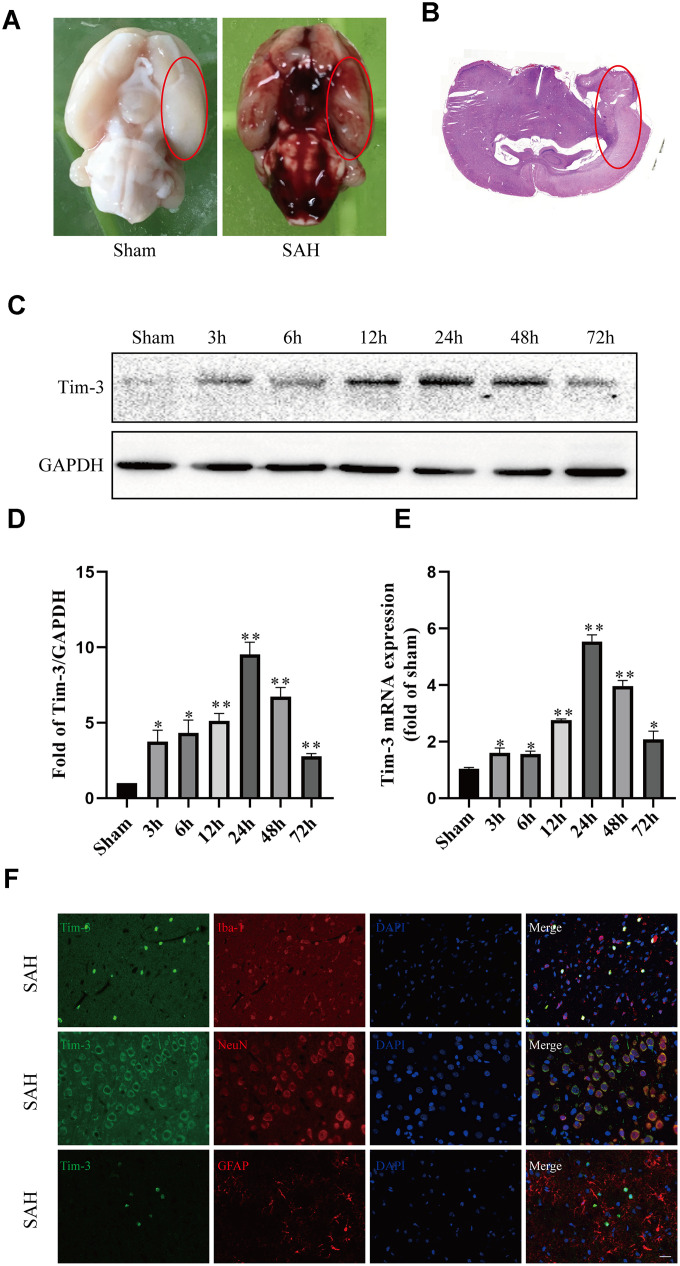
**Representative image of the subarachnoid hemorrhage (SAH) model and endogenous expression of Tim-3 in brain tissue.** Representative images of brains from the sham and SAH groups. (**A**) A schematic indicating the optimal brain region for immunochemical staining, qRT-PCR, and western blotting (red circle). (**B**) Western blot analysis showed Tim-3 protein abundance at 3, 6, 12, 24, 48, and 72 h after SAH. (**C**) Quantification of the Tim-3 protein level. (**D**) Quantification of *Tim-3* mRNA level in the rat brain. (**E**) Representative microphotographs of immunofluorescence staining for Tim-3 and Iba1, NeuN, GFAP. (**F**) All values are presented as means ± SD, n = 6 for each time point per group. *p < 0.05, **p < 0.01 versus sham group. Scale bar = 50 μm.

All rats in different groups are shown in Supplementary Tables 1 and 2. A total of 365 rats were used,72 rats were in the sham group and 246 were in the different SAH treatment groups. A total of 38 rats died, the mortality of rats in the SAH group was 8.92%, while the mortality of rats treats with Tim-3-AAVs was 27.27%. Nine rats were excluded due to SAH grades being < 7; after the exclusion, subsequent rats were added to the groups in order that each group had n = 6. We were primarily interested in the basal cortex of the left hemisphere, and representative images are shown in Figure 1A and Figure 1B. Finally, the SAH grade scores were not significantly different among the SAH groups.

### Temporal patterns of Tim-3 were evaluated after SAH

Western blot and qPCR were performed to assess the protein and mRNA expression, respectively, of Tim-3 at 3, 6, 12, 24, 48, 72 h after SAH in the left cerebral hemisphere. Western blot analysis showed there was a significant increase in endogenous Tim-3 levels in the cortex at 3 h, which peaked at 24 h, and then gradually declined at 48 h, but was still higher than that of the sham group (Figure 1C).

The levels of *Tim*-3 mRNA increased immediately at 3 h after SAH, reached a peak value at 24 h, which almost five times higher than that in the sham group, and then gradually declined at 48 and 72 h (Figure 1D).

### Cellular location of Tim-3

We used double immunofluorescence to determine the cellular localization of Tim-3 at peak expression (at 24 h according to western blotting and qPCR). Double immunofluorescence staining determined that Tim-3 was expressed primarily in microglia and neurons, but was rarely found in astrocytes (Figure 1E).

### Lateral ventricle injection of AAV-Tim-3 increased the expression of Tim-3 and brain neurocyte apoptosis

**Figure 2:**
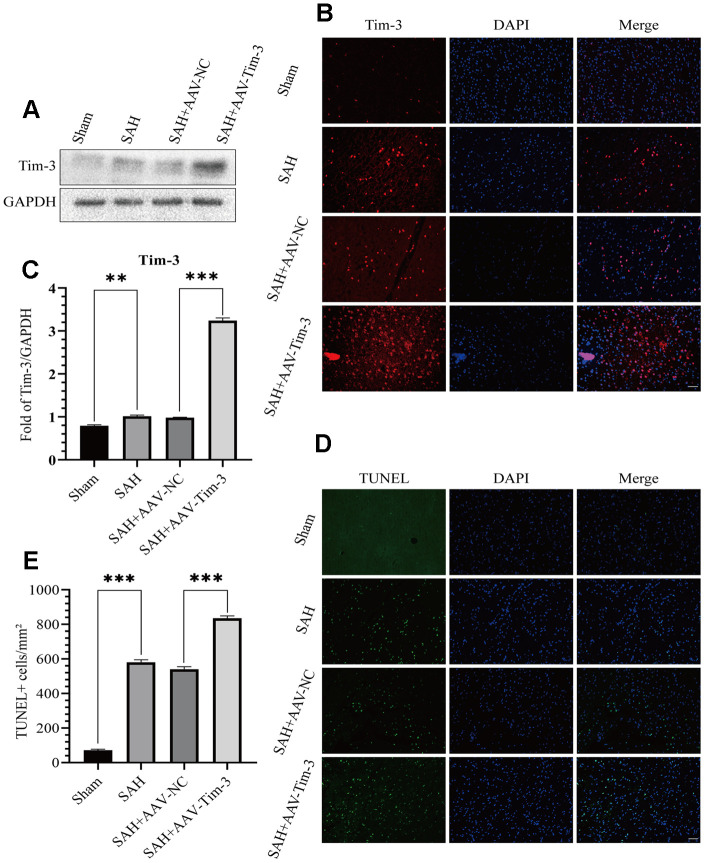
**Effects of AAV-Tim-3 treatment on Tim-3 protein levels and neurocyte apoptosis after SAH.** Western blotting shows that AAV-Tim-3 significantly increased Tim-3 expression (**A**) and quantitative analysis of Tim-3 (**B**). Immunofluorescence staining also verified that AAV-Tim-3 significantly increased Tim-3 expression (**C**). Representative TUNEL staining images (**D**) and quantitative analysis of TUNEL-positive cells (**E**) in the ipsilateral cortex after SAH with AAV-Tim-3 or AAV-NC treatments; n = 6 in each group. Data are expressed as mean ± SD. **p < 0.01, ***p < 0.001.

Lateral intraventricular injection of AAV-Tim3 was performed 3 weeks prior to SAH, and measured 24 h after SAH. Western blotting analysis showed that Tim-3 was noticeably increased after SAH (Figure 2A, 2B), which was verified by immunofluorescence staining (Figure 2C). To further investigate the effects of AAV-Tim-3 treatment on EBI after SAH, TUNEL staining was performed to estimated neurocyte apoptosis. At 24 h after SAH, TUNEL staining revealed that SAH induced a large amount of TUNEL-positive neurocyte in the cortical region of the ipsilateral hemisphere. Administration of AAV-Tim-3 significantly increased the number of TUNEL-positive neurocyte compared with the SAH+AAV-NC group. Furthermore, there was no significant difference between the SAH and SAH + AAV-NC groups (Figure 2D, 2E).

### Lateral ventricle injection of AAV-Tim-3 enhanced EBI, aggravated brain edema, disrupted the BBB, and increased neurological deficits at 24 h after SAH

**Figure 3:**
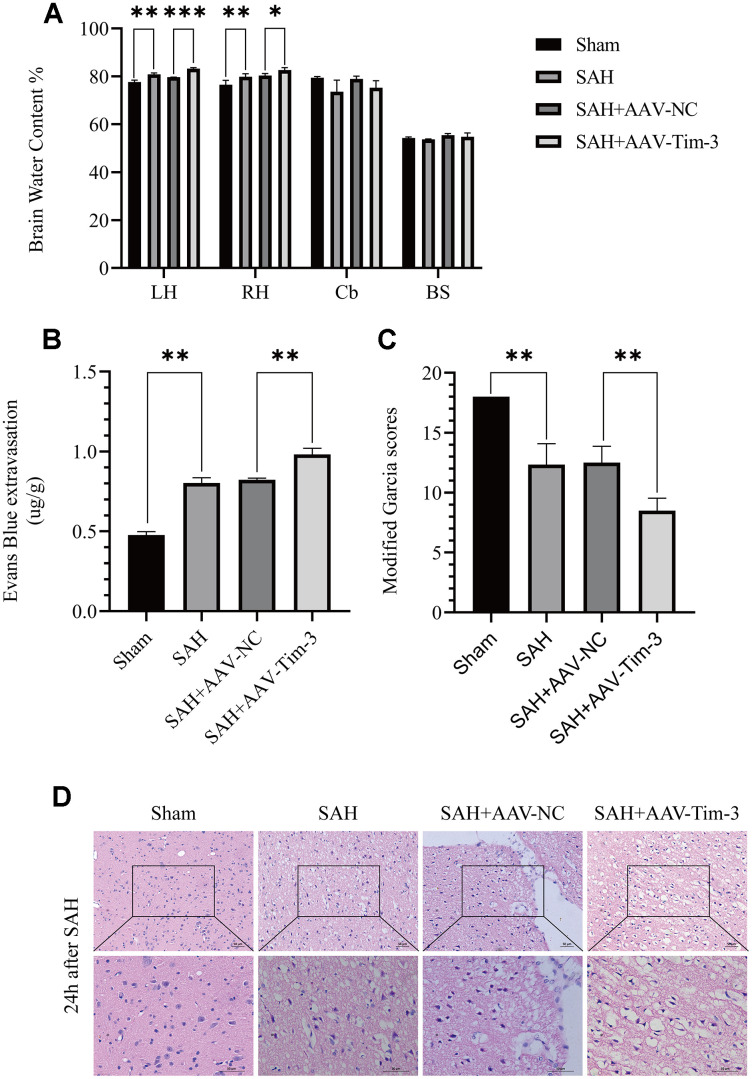
**The effect of AAV-Tim-3 on brain edema, BBB disruption, and neurological function after SAH.** AAV-Tim-3 treatment significantly increased brain water content (BWC) (**A**) and Evans Blue dye extravasation (**B**) at 24 h post-SAH and exacerbated neurological deficits (**C**) (n = 6/group). Representative images of H&E staining (**D**) showing alterations in lesion volume after AAV-Tim-3 treatment. Data are expressed as mean ± SD. *p < 0.05, **p < 0.01, ***p < 0.001. BS, brain stem; Cb, cerebellum; LH, left hemisphere; RH, right hemisphere.

To further explore whether Tim-3 might contribute to SAH induced EBI, brain edema and BBB permeability were measured at 24 h after SAH. The results revealed that the administration of AAV-Tim-3 through intraventricular injection significantly increased brain water content and BBB permeability compared with the SAH+AAV-Tim-3 groups (Figure 3A, 3B). There was no significant difference in brain edema or BBB permeability between the 24 h post-SAH groups and the SAH+AAV-NC groups. In addition, overexpression of endogenous Tim-3 significantly aggravated neurologic deficits at 24 h after SAH (Figure 3C). Furthermore, H&E staining was also used to evaluate brain edema in sections at 24 h post-SAH groups and AAV-Tim-3 treated-groups, the results showed that AAV-Tim-3 aggravated brain histological injury (Figure 3D).

### Lateral ventricle injection of AAV-Tim-3 activated microglia and increased the secretion of inflammatory cytokines by elevating the expression of Tim-3 at 24 h after SAH

**Figure 4:**
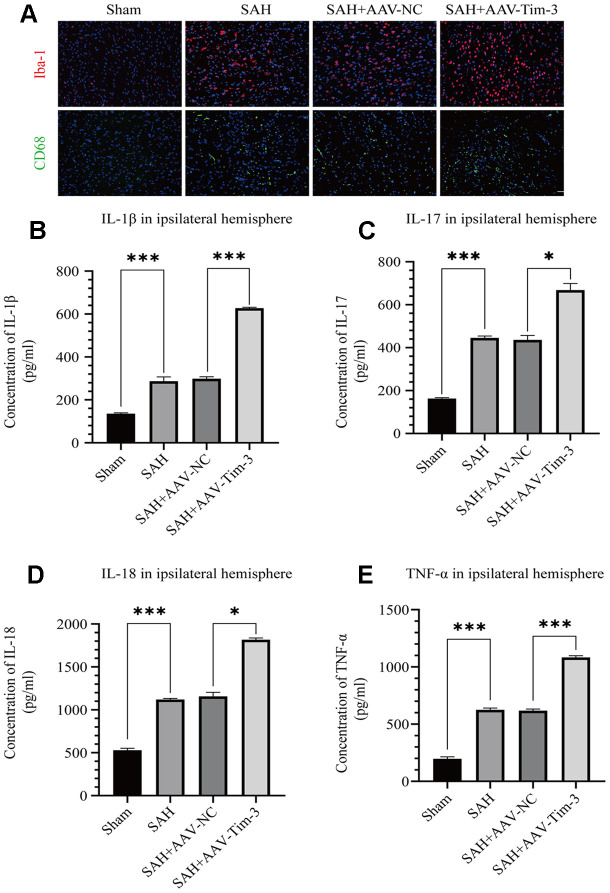
**AAV-Tim-3 treatment stimulated microglial activation and upregulated production of the inflammatory cytokines IL-1β, IL-17, IL-18, and TNF-α at 24 post-SAH.** Representative images of Iba1- and CD68-positive cells in the sham, SAH, SAH+AAV-NC, and SAH+AAV-Tim-3 groups (**A**). AAV-Tim-3 treatment increased the expression of the pro-inflammatory cytokines, IL-1β (**B**), IL-17 (**C**), and IL-18 (**D**), TNF-α (**E**) (n = 6 in each group). Data are expressed as mean ± SD. *p < 0.05, ***p < 0.001. Iba1, ionized calcium binding adapter molecule 1; CD, cluster of differentiation.

Activated microglia are an important source of pro-inflammatory cytokines in the brain. SAH insults significantly increased the expression of Iba-1 when compared with expression in the sham group. Furthermore, with AAV-Tim-3 treatment, Iba-1 and CD68 expression were markedly higher than those in the SAH groups, as revealed by immunofluorescence staining (Figure 4A). To further ascertain the effects of AAV-Tim-3 in downstream inflammatory cytokines release, we measured the release levels of several inflammatory factors in the ipsilateral hemisphere at 24 h post-SAH. Administration of AAV-Tim-3 significantly increased the levels of the proinflammatory cytokines, IL-1β, IL-17, IL-18, and TNF-α compared with the SAH+AAV-NC groups (Figure 4B–4E). In addition, there was no significant difference in brain edema or neurological score between the 24 h post-SAH groups and the SAH+AV-NC groups.

### Lateral ventricle injection of Tim-3 siRNA reduced Tim-3 expression and inhibited neurocyte apoptosis

**Figure 5:**
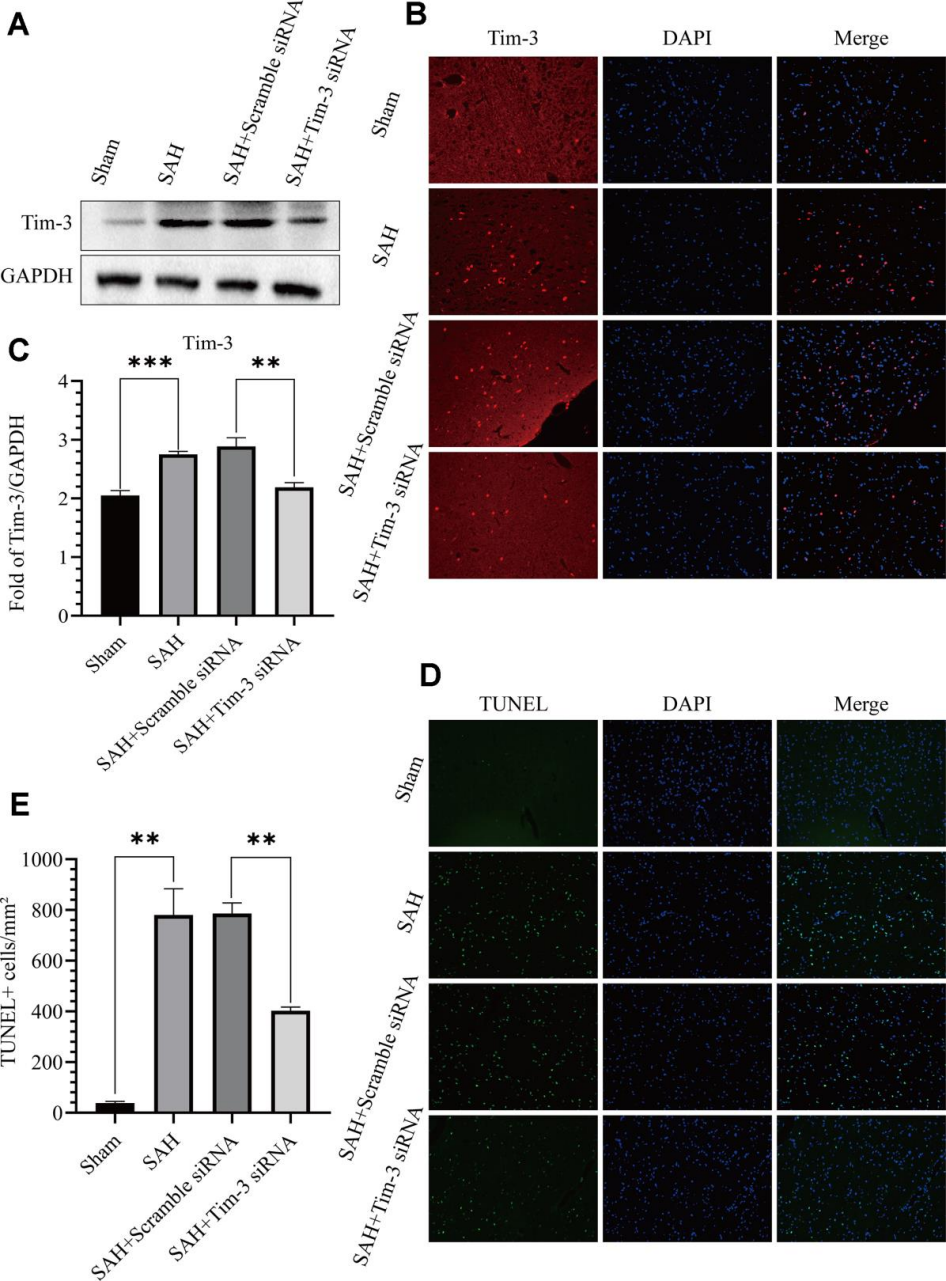
**Effects of Tim-3 knockdown on Tim-3 protein levels and neurocyte apoptosis after SAH.** Western blotting showing that Tim-3 siRNA significantly decreased the expression of Tim-3 (**A**) as assessed by quantitative analysis (**B**). Immunofluorescence staining also verified that Tim-3 siRNA significantly decreased Tim-3expression (**C**). Representative TUNEL staining images (**D**) and quantitative analysis of TUNEL-positive cells (**E**) in the ipsilateral cortex after SAH with Tim-3 siRNA or scramble siRNA treatment (n = 6 in each group). Data are expressed as mean ± SD. **p < 0.01, ***p < 0.001.

To further determine the effect of Tim-3 in EBI after SAH, we detected neurocyte apoptosis and release of related inflammatory factors after knocking down endogenous Tim-3 expression. Tim-3 siRNA was injected intravenously 48 h prior to SAH to knockdown of endogenous Tim-3 expression. Western blotting was used to measure transfection and knockdown efficiency. As shown in Figure 5A–5B, Tim-3 siRNA significantly reduced the expression of endogenous Tim-3 in the brain compared with the SAH groups; however, scramble siRNA had no effect on endogenous Tim-3 expression compared with the SAH groups. The results were also verified by immunofluorescence staining (Figure 5C). TUNEL staining showed that the number of TUNEL-positive cells in the ipsilateral hemisphere in the SAH+Tim-3 siRNA groups were decreased compared with SAH and SAH+Scramble siRNA groups (Figure 5D, 5E).

### Tim-3 knockdown by siRNA alleviated brain edema, reduced BBB permeability, and improved short-term neurological functions and reduced inflammatory cytokine infiltration after SAH

**Figure 6:**
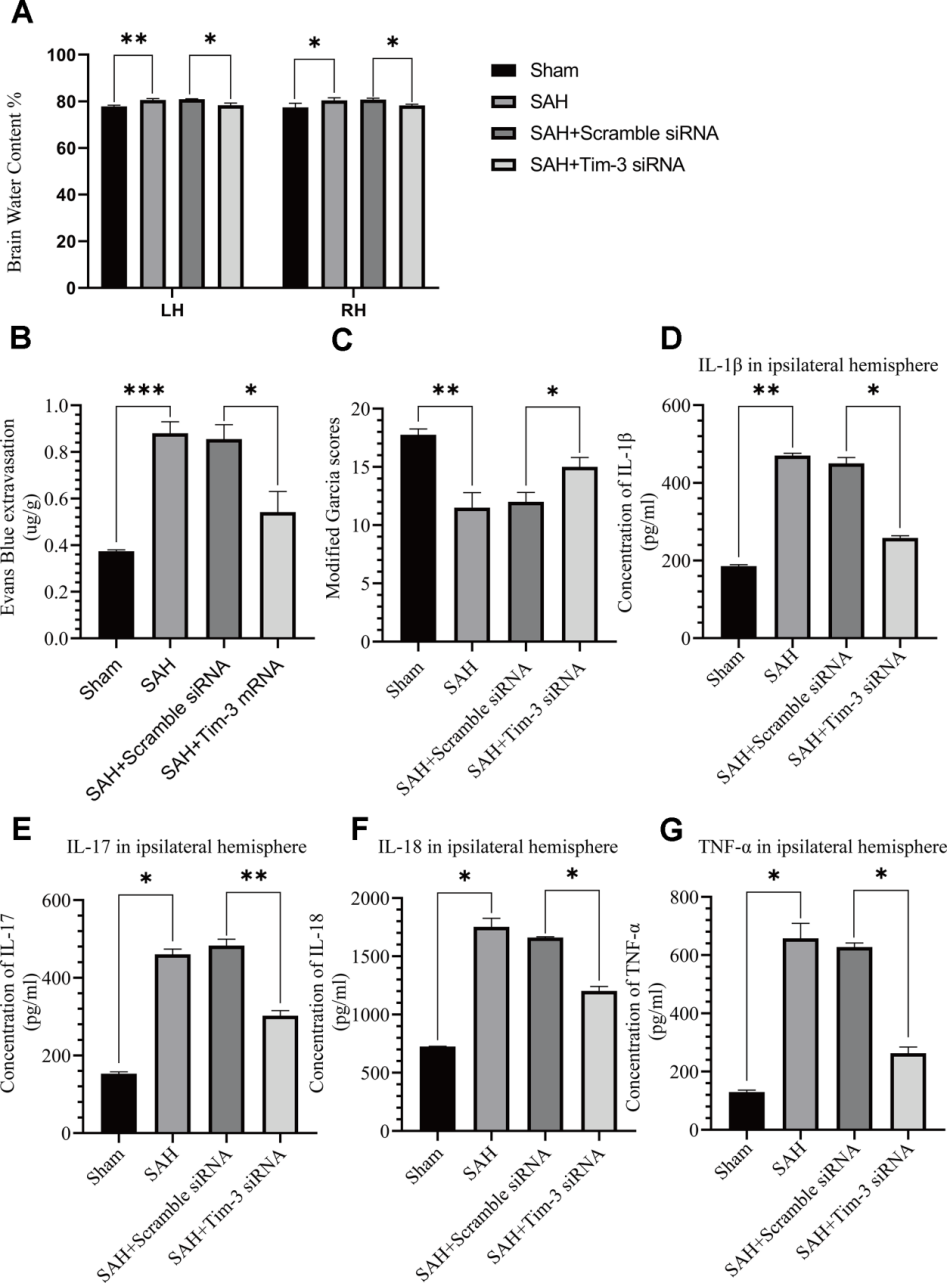
**The effect of Tim-3 knockdown on brain edema, BBB disruption, neurological function, and inflammatory cytokine production after SAH.** Tim-3 siRNA treatment significantly decreased BWC (**A**) and Evans Blue dye extravasation (**B**) at 24 h post-SAH and improved neurological function (**C**) (n = 6 in each group). Tim-3 siRNA treatment decreased the expression of the inflammatory cytokines, IL-1β (**D**), IL-17 (**E**), and IL-18 (**F**) TNF-α (**G**). Data are expressed as the mean ± SEM.*p < 0.05,**p < 0.01, ***p < 0.001.

The effects of Tim-3 on BBB permeability, brain edema, neurologic function, and brain inflammation were determined at 24 h after SAH. Knockdown of *Tim*-3 expression significantly alleviated brain edema (Figure 6A), reduced BBB permeability (Figure 6B), and improved short-term neurological functions (Figure 6C) at 24 h after SAH. *Tim*-3 knockdown significantly increased the levels of proinflammatory, IL-1β, IL-17, IL-18, and TNF-α compared with the SAH and SAH+Scramble siRNA groups. There was no significant difference in inflammatory cytokine infiltration between the 24 h post-SAH groups and the SAH+Scramble siRNA groups (Figure 6D–6G).

### Effect of Tim-3 on Nrf2 and HMGB1 expression and distribution after SAH

**Figure 7:**
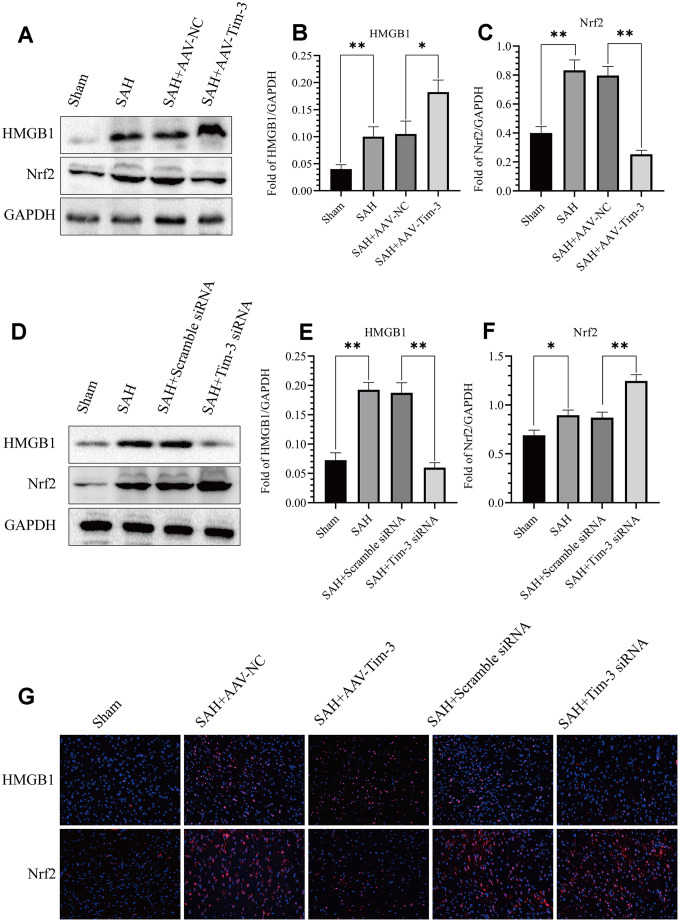
**Effect of AAV-Tim-3 or Tim-3 siRNA on the expression of HMGB1 and Nrf2.** Western blot showing that AAV-Tim-3 significantly increased the expression of HMGB1 and reduced the expression of Nrf2, (**A**) as assessed by quantitative analysis of HMGB1 (**B**) and Nrf2 (**C**). Tim-3 siRNA treatment significantly decreased the expression of HMGB1 and increased the expression of Nrf2, (**D**) as assessed by quantitative analysis of HMGB1 (**E**) and Nrf2 (**F**) in the left hemisphere at 24 h post-SAH (n = 6 in each group). Data are expressed as the mean ± SEM. *p < 0.05,**p < 0.01. Representative microphotographs of immunofluorescence staining for HMGB1 and Nrf2 (**G**) following AAV-Tim-3 or Tim-3 siRNA treatments in the left cerebral cortex at 24 h post-SAH. Scale bar = 50 μm.

The expression and distribution of Nrf2 and HMGB1 were identified by western blot analysis and immunofluorescence. Results of western blot analysis showed that SAH insults induced a significant increase in Nrf2 and HMGB1 expression compared with that in the sham groups. However, treatment with AAV-Tim-3 significantly reduced the levels of Nrf2 and increased the levels of HMGB1 compared with SAH+AAV-NC groups (Figure 7A–7C). Compared with the SAH+Scramble siRNA groups, Tim-3 siRNA pretreatment decreased the expression of HMGB1, while increasing the expression of Nrf2 (Figure 7D–7F). To further verify these results, immunofluorescence staining was used to determine the expression levels of HMGB1 and Nrf2 in brain tissue sections. Nrf2 and HMGB1 were significantly increased in the SAH and SAH+AAV-NC groups. In the AAV-Tim-3 groups, Nrf2 expression was decreased, while HMGB1 expression was significantly increased. Finally, Tim-3 siRNA pretreatment decreased the expression of HMGB1, while it increased the expression of Nrf2, consistent with western blot results (Figure 7G).

### NK-252 abolished the inflammation effects of Tim-3 by increasing Nrf2 expression

**Figure 8:**
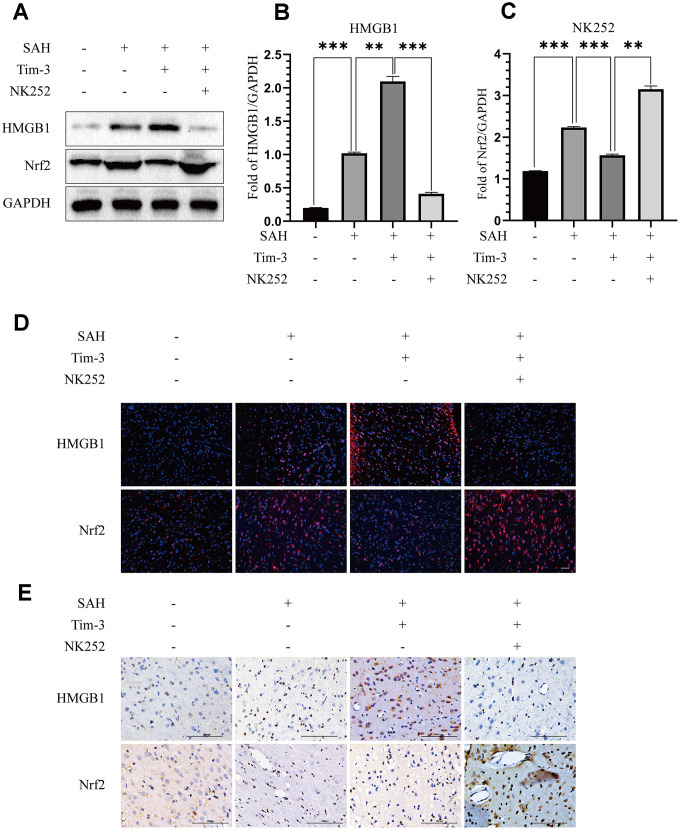
**NK252 treatment reversed the effect of Tim-3 on the expression of HMGB1 and Nrf2.** Compared with the SAH+AAV-Tim-3+DMSO groups, western blotting data indicated that NK252 significantly decreased the expression of HMGB1 and increased the expression of Nrf2 (**A**) as assessed by quantitative analysis of HMGB1 (**B**) and Nrf2 (**C**) in the left hemisphere at 24 h post-SAH(n = 6 in each group). Data are expressed as the mean ± SEM.**p < 0.01,***p < 0.001. Representative microphotographs of immunofluorescence staining for HMGB1 and Nrf2 (**D**). Scale bar = 50 μm. Immunohistochemical staining of HMGB1 and Nrf2 (**E**) in the left cerebral cortex at 24 h post-SAH. Scale bar = 100 μm.

**Figure 9:**
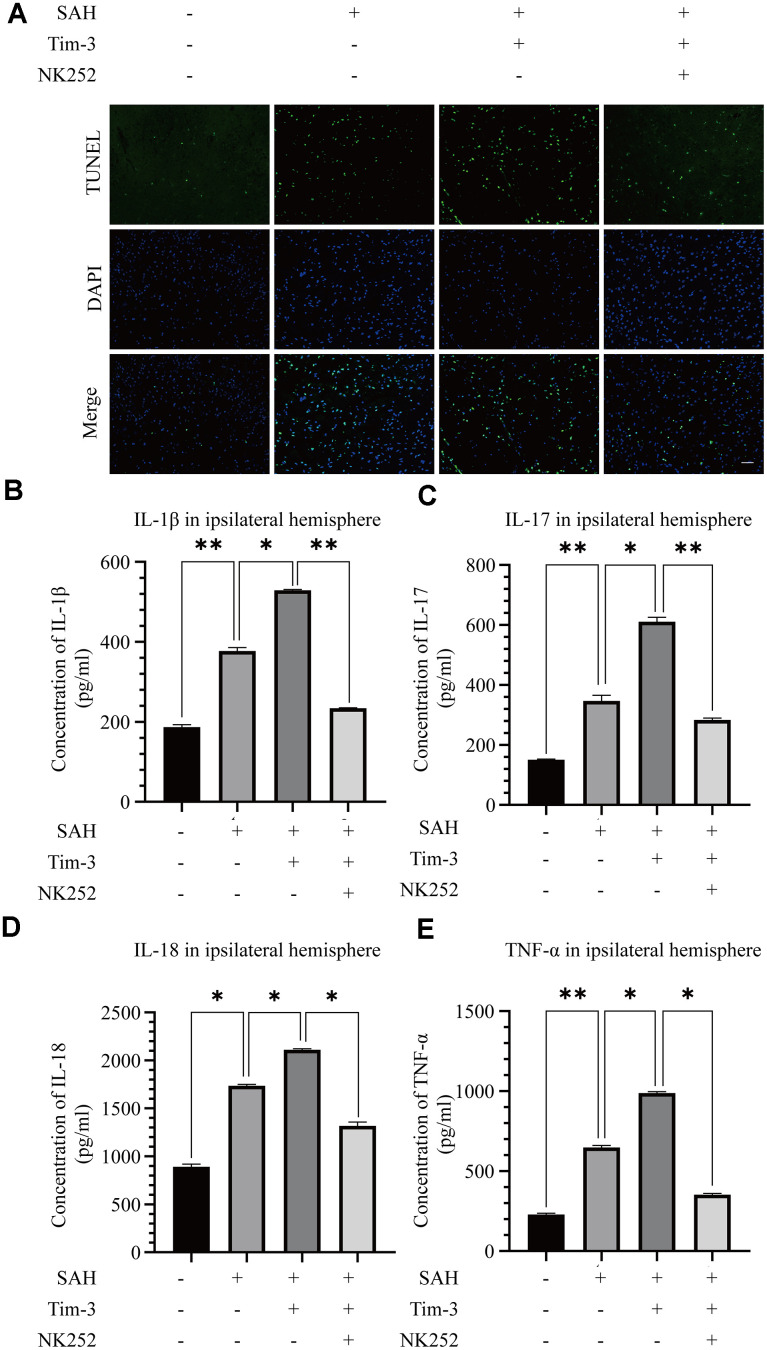
**NK252 treatment abolished neurocyte apoptosis and the inflammatory effects associated with Tim-3.** TUNEL staining showing that NK252 treatment significantly decreased TUNEL-positive neurocyte (**A**) and the expression of the pro-inflammatory cytokines, IL-1β (**B**), IL-17 (**C**), and IL-18 (**D**) TNF-α (**E**) (n = 6 in each group). Data are expressed as the mean ± SEM.*p < 0.05,**p < 0.01. Scale bar = 50 μm.

**Figure 10:**
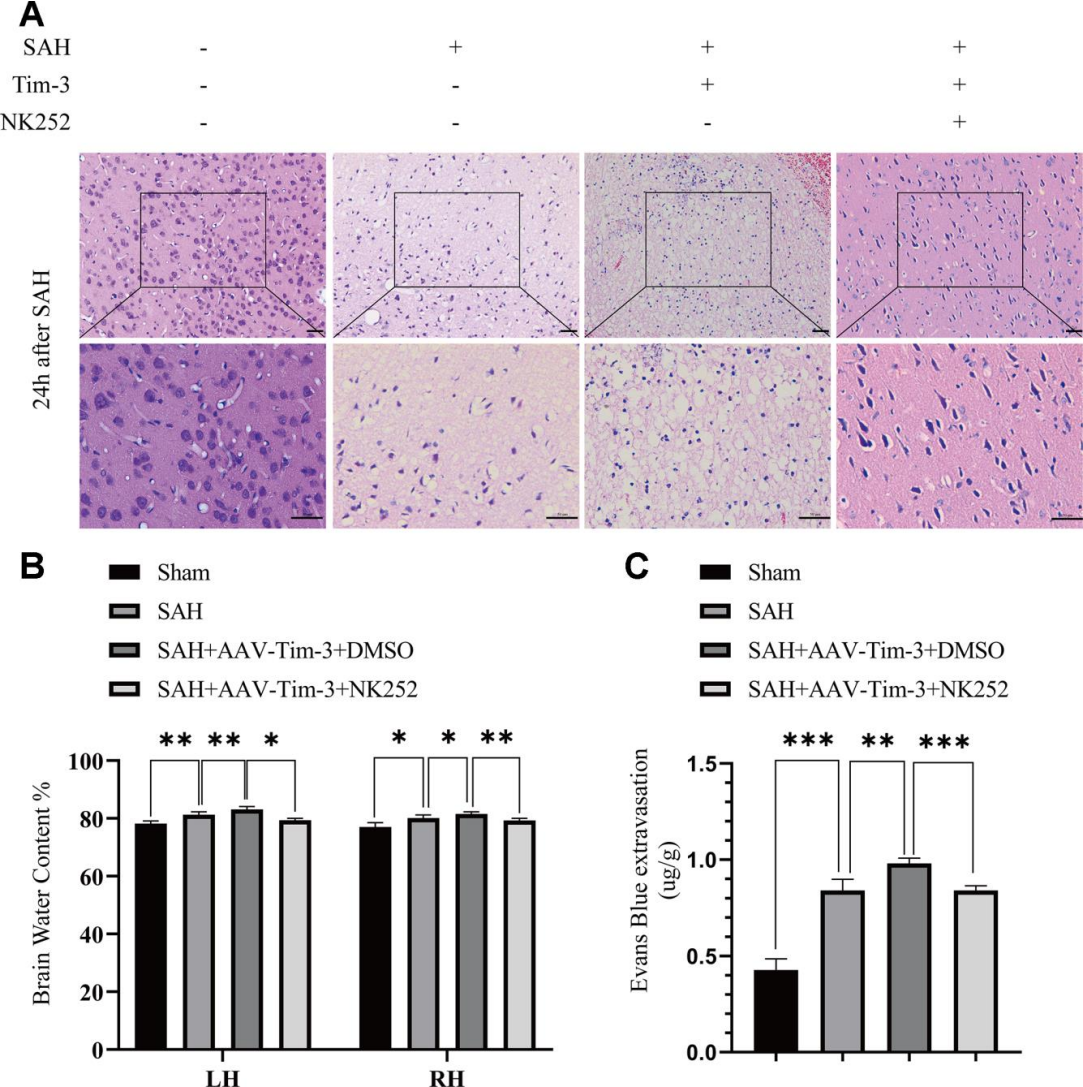
**Administration of NK252 reversed the brain edema, BBB disruption associated with Tim-3 after SAH.** Representative images of H&E staining showing alterations in lesion volume after NK252 treatment (**A**). NK252 treatment significantly decreased BWC (**B**) and Evans Blue dye extravasation (**C**) at 24 h post-SAH(n = 6 in each group). Data are expressed as the mean ± SEM.*p < 0.05,**p < 0.01, ***p < 0.001. Scale bar = 50 μm.

To further evaluate whether Tim-3 promotes HMGB1 and plays a pro-inflammatory role by inhibiting Nrf2 expression, NK-252, an Nrf2 specific agonist, was used to explore the potential mechanism of Tim-3-related neuroinflammation. NK252 (dissolved in DMSO, 50 μg/kg,) was administrated intracerebroventricularly to rats at 24 h prior to SAH. Results of western blotting showed that protein levels of Nrf2 were upregulated after treatment with NK252 compared with the SAH+AAV-Tim-3+DMSO group, while the expression of HMGB1 was obviously decreased (Figure 8A–8C); a result confirmed by immunohistochemistry (Figure 8D) and immunofluorescence (Figure 8E). In addition, the increase in TUNEL-positive neurocyte was reversed following treatment with NK252 (Figure 9A). Administration of NK252 significantly decreased the secretion of proinflammatory factors, such as IL-1β, IL-17, IL-18, and TNF-α compared with the SAH+AAV+DMSO group (Figure 9B–9E). Furthermore, H&E staining was also used to evaluate brain edema in sections at 24 h post-NK252 groups and AAV-Tim-3 treated-groups, the results showed that NK252 treatment significantly alleviated brain histological injury (Figure 10A). In addition, NK252 treatment significantly mitigated brain edema (Figure 10B), reduced BBB permeability (Figure 10C) at 24 h after SAH.

## DISCUSSION

In this study, we revealed the potential role of Tim-3-induced aggravation of EBI and neuroinflammation in a rat model of SAH, which involved the regulation of Nrf2 and HMGB1. Our data also revealed that Tim-3 was upregulated in the cerebral cortex in a time-dependent manner and peaked at 24 h after SAH. Furthermore, endogenous Tim-3 was expressed primarily in microglia and neurons but was rarely found in astrocytes. Interestingly, this result was in contrast to the work of Chen et al., in which they found Tim-3 was located exclusively in microglia in intracranial hemorrhage (ICH) rat model [22]. In addition, in our results, Tim-3 was expressed in the nucleus of microglia, while in neurons it was expressed in the cell membrane. We speculated that Tim-3 may have different roles in these two different cell types. To further determine the role of Tim-3 in EBI following SAH, we generated a rat SAH model. Increased expression of Tim-3 by transfection with AAV significantly aggravated neurological dysfunction, exacerbated brain edema, and increased BBB permeability after SAH *in vivo*. Furthermore, overexpression of Tim-3 also promoted neurocyte apoptosis and the secretion of inflammatory cytokines after SAH. In contrast, knockdown of endogenous Tim-3 with siRNA decreased the secretion of inflammatory cytokines in the brain after SAH, improved neurological outcomes, ameliorated brain edema, and decreased BBB permeability after SAH. To identify possible signaling pathways by which Tim-3 aggravates the neuroinflammatory response, signaling proteins associated with Tim-3 in brain tissue after SAH were investigated. Results showed that the neuroinflammatory effect of Tim-3 may involve the regulation of Nrf2 and HMGB1, important elements in the pathogenesis of the neuroinflammatory response. We showed that overexpression of Nrf2 significantly reversed the neuroinflammatory effects of Tim-3 and attenuated brain injury. Accordingly, we demonstrated that Tim-3 augmented the expression of HMGB1 and aggravated neuroinflammation by suppressing the expression of Nrf2 after acute SAH in rats.

As previously reported, both clinical and experimental results of SAH showed that the inflammatory response induces EBI leading to BBB leakage, brain edema, neurocyte death, and neurological dysfunction. In addition, patients with elevated inflammation had poorer clinical outcomes. However, the definitive molecular mechanism of the pathological process is still not fully understood. Here, we conducted a preliminary study on the potential role of Tim-3, a novel immunomodulatory molecule, in SAH.

As an immuno-regulation molecule, Tim-3 was found to be expressed in both adaptive and innate immune cells, including Th1 cells, Tc1 cells, microglia, monocytes, mast cells, dendritic cells and natural killer cells [18, 20, 29]. Indeed, it has been found to play complex roles in immune regulation in several inflammatory diseases. Previous reports have shown that Tim-3 is associated with several CNS diseases, but plays a different role in different diseases. For example, Tim-3 was shown to play a crucial role in the maintenance of peripheral tolerance in experimental autoimmune encephalomyelitis (EAE). Specifically, abolishing Tim-3 signaling during CD8+ T cell–mediated EAE exacerbated the disease [28]. Moreover, recent studies reported that increased Tim-3 expression in peripheral blood mononuclear cells in ischemic stroke patients and in brain tissue of ischemia-reperfusion mice positively correlated with plasma IL-17 and TNF-α levels [25]. Furthermore, Tim-3 played an important role in the neuroinflammatory response induced by cerebral hemorrhage, aggravating the inflammatory response after cerebral hemorrhage [30]. Both clinical studies and animal experiments have demonstrated that increased Tim-3 expression is associated with poor prognosis after cerebral hemorrhage [22, 30, 31]. These results collectively suggest that Tim-3 plays a role in the response of innate and adaptive immunity in the brain. However, it is unknown whether Tim-3 is involved in the pathogenesis of inflammation following SAH. Here, our results provide evidence that Tim-3 is involved in the inflammatory response to EBI following SAH; specifically, Tim-3 was significantly increased in brain tissue of rats with SAH. Furthermore, our results revealed that Tim-3 overexpression significantly increased the inflammatory response and aggravated cerebral edema and neurological deficits in rats after SAH. Interestingly, we found that lateral ventricle injection of AAV-Tim-3 significantly aggravated the apoptosis of nerve cells. However, our results are contrary to previous report that Tim-3 inhibition increased cell apoptosis in HL-60 cell line [32]. In this case, it may depend on the cell type as well as inflammatory effects of Tim-3, the exact mechanism needs to be explored further. In addition, Tim-3 reduced Nrf2 expression in our result, a result that was consistent with the work of Wang et al., which demonstrated that Tim-3 may decrease Nrf2 protein levels by inducing Nrf2 ubiquitination and degradation by the proteasome complex [33].

Nrf2, a protein essential for protection against oxidative/xenobiotic stresses [34, 35], has been known to play an important role in anti-oxidative stress and anti-inflammation in the liver, kidney, lung, and brain [13, 36, 37]. In the brain, Nrf2 is expressed in microglia, neurons, and astrocytes, acting as an anti-inflammatory agent in many CNS diseases, such as cerebral hemorrhage and cerebral ischemia [38, 39]. Importantly, Nrf2 has been shown to contribute to the pathophysiological progression of SAH by regulating oxidative stress and the inflammatory response in rats [13, 14]. Hence, in agreement with this observation, knockdown of Nrf2 expression may be closely related to severe inflammation and poor prognosis in SAH.

On the other hand, increasing evidence shows that HMGB1, a member of the DAMP family, functions as a novel pro-inflammatory cytokine. HMGB1 is actively secreted by inflammatory cells or released passively by necrotic cells [40, 41]. Previous studies have demonstrated that the levels of HMGB1 were significantly increased within 2 h after SAH [10]. Meanwhile, increased levels of HMGB1 have also been shown to stimulate inflammatory cytokine secretion, such as IL-1β and TNF-α, in experimental SAH models [42]. Therefore, the expression of HMGB1 is crucial in the inflammatory response after SAH.

Several studies have shown that Nrf2 can play an anti-inflammatory role by reducing the expression of HMGB1 in some inflammatory diseases. For example, Kim et al. showed that ascorbic acid reduced HMGB1 expression by activating the Nrf2 signaling pathway following sepsis [43]. Other literature supported Nrf2-mediated suppression of HMGB1 in neuropathic pain, acute lung injury, and renal ischemia reperfusion and intestinal ischemia-reperfusion [44–46]. In addition, Chang et al. previously reported that tetramethylpyrazine elevated Nrf2/HO-1 expression and inhibited HMGB1/TLR4 expression, promoted endogenous anti-inflammatory defense capacity, and attenuated pro-inflammatory responses in cerebral ischemia [47].

Regarding the possible mechanisms underlined in Tim-3-related inflammation in SAH, we discovered that treatment with AAV-Tim-3 effectively increased the expression of Tim-3 in the ipsilateral hemisphere following SAH, while it significantly inhibited Nrf2 expression and increased HMGB1 expression and the secretion of inflammatory cytokines, including IL-1β, IL-17, IL-18, and TNF-α compared with the SAH+AAV-NC group. *In vivo* knockdown of Tim-3 by siRNA significantly reduced HMGB1 expression and the secretion of related pro-inflammatory factors, but significantly increased Nrf2 expression. In order to further explore whether Tim-3 promotes HMGB1 expression and plays a pro-inflammatory role by inhibiting Nrf2 expression in SAH, we administered NK252, an Nrf2 specific agonist, to SAH rats 3 weeks prior to AAV-Tim-3 injection. The results indicated that the administration of NK252 abolished the inflammatory effects of Tim-3 by promoting Nrf2 expression, inhibiting HMGB1 expression, and reducing pro-inflammatory cytokine secretion. Taken together, these results suggest that Tim-3 overexpression appears to deteriorate neuroinflammatory and neurocyte apoptosis after subarachnoid hemorrhage through the Nrf2/HMGB1 signaling pathway in rats.

In addition, there were several limitations to our study that need to be addressed. Firstly, the inflammatory response is one of the important mechanisms of EBI after SAH, and the subsequent related oxidative stress, BBB disruption, vascular brain edema, and intracranial pressure further contribute to the injury associated with EBI [48]. Here, we only studied aggravation of the neuroinflammatory response by inhibiting Nrf2 expression but did not discuss whether Tm-3 was involved in the pathophysiological process of EBI by influencing oxidative stress or other factors. Secondly, we only demonstrated that Tim-3 promoted HMGB1 expression and aggravated the neuroinflammatory response after SAH by inhibiting Nrf2, but we did not test other related pathways associated with Tim-3-mediated neuroinflammation. Hence, further investigations are needed to fully clarify the precise mechanisms involved. Finally, our study mainly focused on the role of Tim-3 in the early stage of SAH injury. The potential long-term roles of Tim-3 on neuroinflammation following a brain injury are not confirmed and should to be explored in future studies.

## CONCLUSIONS

In summary, this study indicated that Tim-3 is a molecular player that links neuroinflammation and brain damage after SAH. We revealed that Tim-3 aggravates neuroinflammation and deteriorates neurocyte apoptosis through the Nrf2/HMGB1 signaling pathway after acute SAH in rats, which may expand application in the future development of effective therapeutic approaches against cerebrovascular diseases.

## MATERIALS AND METHODS

### Animals

Sprague-Dawley rats weighing 280–320 g and ~8 weeks old, were purchased from the Animal Experiment Center of Southern Medical University (Guangzhou, China). All rats were fed and housed in a quiet environment (indoor temperature ~22 ± 1 °C, humidity 40%–60%) with a 12:12 dark/light cycle.

### Experimental design

**Figure 11:**
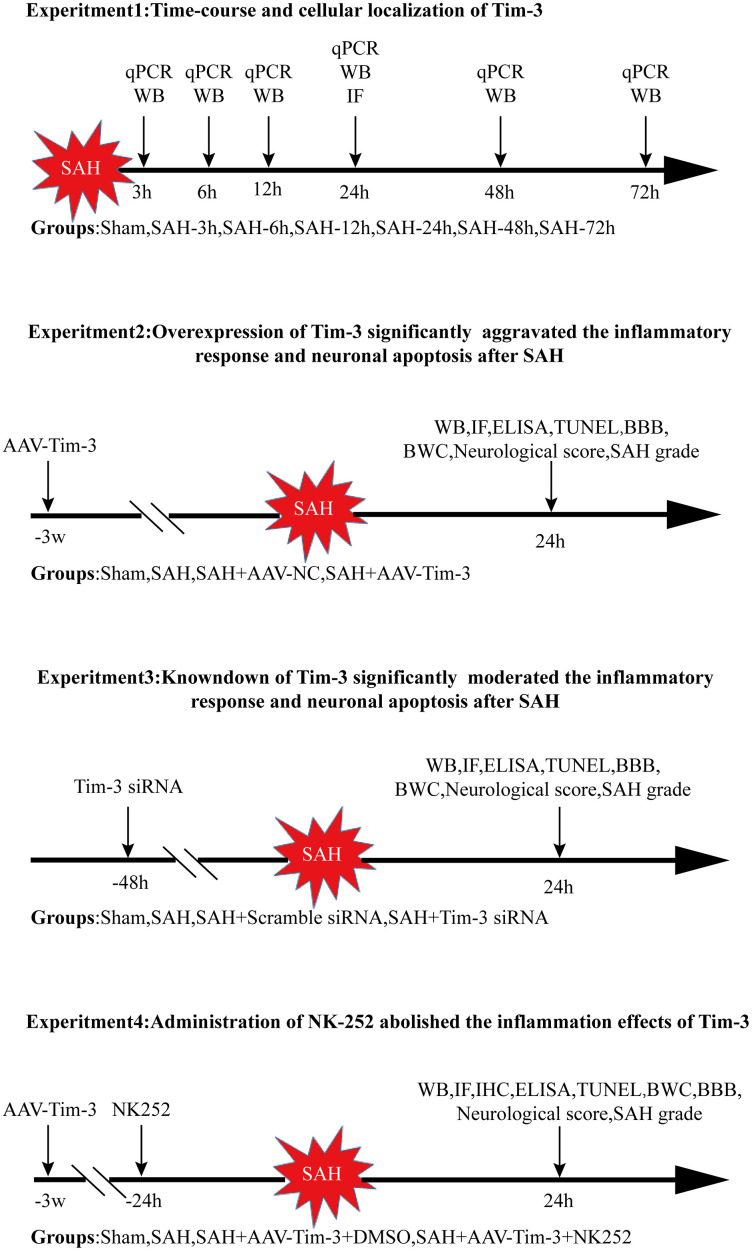
**Experimental design and animal group classification.** SAH = subarachnoid hemorrhage; qRT-PCR = quantitative real-time polymerase chain reaction WB = western blotting; BWC = brain water content; IF = Immunofluorescence staining; IHC = immunohistochemical staining; ELISA = enzyme-linked immunosorbent assay; TUNEL = terminal deoxynucleotidyl transferase-mediated biotinylated-dUTP nick-end labeling.

The experiments were performed as follows (Figure 11).

### Experiment 1

Forty-eight male rats were randomly divided into seven groups; sham and SAH 3, 6, 12, 24, 48, and 72 h post operation. The ipsilateral cerebral cortex of six rats from each group was collected for quantitative real-time polymerase chain reaction (RT-qPCR) and western blotting. Additionally, the cellular location of Tim-3 was evaluated by double immunofluorescence staining at 24 h post-SAH.

### Experiment 2

In order to explore the role of Tim-3 on EBI after SAH, 90 rats were randomly divided into sham, SAH, SAH+adeno-associated virus (AAV)-negative control (NC), and SAH+AAV-Tim-3 groups. AAV-NC or AAV-Tim-3 was administrated by injection into the lateral ventricle 3 weeks before SAH. All rats were sacrificed 24 h after SAH according to the results of the first experiment. The expression of Nrf2 and HMGB1 was detected by western blotting, immunohistochemistry, and immunofluorescence. Neurocyte apoptosis was performed by TUNEL staining. Inflammatory cytokine levels were measured by ELISA. Neurological scores and brain water content were also analyzed.

### Experiment 3

To further determine the change of inflammation in SAH following Tim-3 knockdown, 90 rats were randomly divided into sham, SAH, SAH+ scramble small interfering (si)RNA, and SAH+Tim-3 siRNA groups. siRNA administration was conducted at 48 h prior to SAH. We detected the protein expression of Nrf2 and HMGB1, the level of neurocyte apoptosis, and the release of related inflammatory cytokines. Neurological scores and brain water content were also analyzed.

### Experiment 4

To further evaluate whether Tim-3 induces HMGB1 expression and plays a pro-inflammatory role by inhibiting Nrf2 expression, N-[5-(2-Furanyl)-1,3,4-oxadiazol-2-yl]-N'-(2-pyridinylmethyl) urea (NK252) was administrated intraventricularly. 90 rats were randomly divided into sham, SAH, SAH+AAV-Tim-3+DMSO, and SAH+AAV-Tim-3+NK252 groups. We detected the expression of Nrf2 and HMGB1 and the release of related inflammatory cytokines.

### SAH models

The SAH model was established in rats *via* intravascular puncture, as previously described [49]. In brief, rats were placed on the operating table in the supine position under deep anesthesia with 1% pentobarbital sodium (40 mg/ kg, i.p.). The left common carotid artery (CCA), external carotid artery (ECA), and internal carotid artery (ICA) were isolated, a 4-0 sharp nylon thread was inserted into the ECA and passed through the bifurcation of the CCA into the ICA and continued into the middle cerebral artery until a significant breakthrough was felt. The thread was then immediately withdrawn to re-initiate blood flow into the ICA. Rats in the sham group underwent the same procedure without puncturing blood vessels.

### SAH grade

SAH grade was assessed by experimenters blinded to the procedure at 24h after SAH. After the rats were euthanized and the entire brain was removed, the basal cistern was immediately photographed and was divided into six segments, as previously described [49]. Each area was assigned to an experimenter blinded to the procedure and a score from 0 to 3 was given, depending on the amount of blood clotting. The total score was obtained by summing the score of all six segments, details are shown in Additional File 1. The total score ranged from 0 to 18, and rats with a SAH grade ≤ 7 at 24 h after SAH were excluded from the study.

### Neurological score

The modified Garcia scoring system was used to assess the neurological score at 24 h after SAH [50, 51]. Specifically, six test subscores were evaluated, including spontaneous activity, spontaneous movement of all limbs, forelimb outstretching, climbing, the touch of the trunk, body proprioception, and response to vibrissae touch. Lower scores indicated more severe neurological deficits.

### Brain water content (BWC)

The brain was quickly removed at 24 h after SAH and immediately divided into four parts: the left hemisphere, right hemisphere, brainstem, and cerebellum, and was immediately weighed to obtain the wet weight and was then dried for 72 h at 105 °C to obtain the dry weight. The percentage of brain water content was calculated as follows: [(wet weight dry weight)/wet weight] × 100%.

### Blood-Brain Barrier (BBB) disruption

BBB disruption was assessed on the basis of Evans blue dye spillover, as previously described [52]. Briefly, Evans blue (EB) dye (2%, 5 ml/kg) was administered into the left femoral vein and circulated for 2 h. Brain tissue was then collected under deep anesthesia and rapidly divided into the left and right hemispheres. The hemispheres were weighed and homogenized in 50% trichloroacetic acid solution (2 mL), then centrifuged at 15000 g for 30 min. The supernatant was added to anhydrous ethanol at a volume ratio of 1:3 and incubated overnight at 4 °C. After centrifugation (15,000 g for 30 min), the resultant supernatant was detected by a multifunctional enzyme marker (excitation light wavelength: 620 nm). The fluorescence density value of the mixture of supernatant and anhydrous alcohol was obtained. EB dye leakage was expressed as μg EB dye/g brain tissue.

### Intracerebroventricular injection

For overexpression of Tim-3 *in vivo*, recombinant AAV (rAAV2/9), Tim-3-rAAV (r-AAV-CMV-TIM-3-P2A-EGFP-WPRE-bGH-polyA, AAV2/9,3.12e + 12 vg/ml), or scrambled NC rAAV (rAAV-CMV-EGFP-pA, AAV2/9,6.20e + 12 vg/ml) was used. All rAAVs were produced by BrainVTA, Wuhan, China. The SAH model was established 3 weeks after AAV injection.

For overexpression of Nrf2 *in vivo*, we used the Nrf2 specific agonist, NK252 (MCE, Monmouth Junction, New Jersey, USA). NK252 was dissolved into dimethyl sulfoxide (DMSO)(Sigma-Aldrich, St. Louis, MO, USA) and was injected (5 ug/ul) into the lateral ventricle 24 h prior to SAH. The sham group followed the same protocol with an equal volume of DMSO.

To lower the expression of Tim-3 *in vivo*, 5 ul siRNA (Sangong Biotech, Shanghai, China) was injected into lateral ventricles 48 h prior to SAH. The same volume of Scr siRNA (Sangong Biotech, Shanghai, China) was used as a negative control. To ensure knockdown efficacy, we mixed the three target sequences (Table 1).

**Table 1:** Sequences for siRNA used in this study.

**Sequences name**	**Sequence**	
Tim-3-rat-157	sense	GGUCGGGAAGAAUGCCUAUTT
	antisense	AUAGGCAUUCUUCCCGACCTT
Tim-3-rat-415	sense	CCCUGGCCCAAUGAAUGAUTT
	antisense	AUCAUUCAUUGGGCCAGGGTT
Tim-3-rat-748	sense	GCAGGAUUUGAGUCUUAUUTT
	antisense	AAUAAGACUCAAAUCCUGCTT
Scramble siRNA	sense	UUCUCCGAACGUGUCACGUTT
	antisense	ACGUGACACGUUCGGAGAATT

Rats were anesthetized with 1% pentobarbital sodium (40 mg/kg, i.p.) and placed on a stereotactic apparatus. The needle of a 2 uL or 10 uL Hamilton syringe (Hamilton Company, Switzerland) was inserted into the left lateral ventricle through a burr hole at the following coordinates relative to bregma: 1.5 mm posterior, 1.0 mm lateral, and 4.5 mm below the horizontal plane of bregma. The agentia (including AAV, NK252, and siRNA) was inserted at a rate of 1 ul/min. After the injection, the needle stays in place for 5 minutes, and then retract the needle in three times, each time withdrawing 1.5 mm and staying there for 3 minutes. Finally, the borehole was sealed with sterile bone wax.

### Western blotting

As previously described, Western blotting was performed from protein isolated from the left cerebral cortex [53]. Rats were anesthetized and transcardially perfused with 250 mL pre-cooled phosphate buffered saline (PBS; 0.1 M, pH 7.4). Brains were then removed and rapidly divided into the left and right hemispheres The left cerebral cortex (50 mg) was homogenized using a polypropylene pestle in 500 uL of RIPA Lysis Buffer (Beyotime Biotechnology, Shanghai, China) and the total protein concentration was measured using a bicinchoninic acid protein assay kit (Genecopoeia, Rockville, MD, USA) according to the manufacturer’s instructions. Equal amounts of the sample protein (50 mg) were separated by a 10% SDS-PAGE gel (Beyotime) and transferred to polyvinylidene fluoride membranes. Membranes were blocked in 5% nonfat milk dissolved in tris-buffered saline with 0.1%Tween-20 (TBST) for 2 h at room temperature, and were incubated overnight at 4 °C with the following primary antibodies: rabbit anti-Tim-3 (1:1000, ab185703, Abcam), rabbit anti-Nrf2 (1:1000, ab89443, Abcam), or rabbit anti-HMGB1 (1:1000,3935S, Cell Signaling Technology). Membranes were then washed three times for 10 min each in TBST, incubated with horseradish peroxidase-conjugated secondary antibody (1:10000, ANR02-1, NeoBioscience) for 1 h at room temperature, and washed with TBST three times for 10 min each. Afterward, antibodies were detected by using the ECL Western blotting detection system (Millipore, Darmstadt, Germany). The expression of each protein was measured with Image J software (Image J 1.5, National Institutes of Health, Bethesda, MD, USA). Rabbit anti-GAPDH (1:2000, ab9485, Abcam) was used as an internal control.

### Hematoxylin and Eosin (H&E) staining

At 24 h post-SAH, rats underwent cardiac perfusion after deep anesthesia with 250 mL pre-cooled PBS (0.1 M, pH 7.4) followed by 500 mL 4% paraformaldehyde, as described previously. The brains were removed and soaked in the same fixative for 24-48 h at 4 °C then embedded in paraffin wax and cut into a series of coronal slices. Sections (~4 μm) were dewaxed and rehydrated, and were counterstained with hematoxylin for 5 min and eosin for 10 s, rinsed with running water for 3 min, and sealed with neutral resin. The slices were viewed and captured on a optical microscope (Leica-DM2500, Wetzlar, Germany).

### Immunofluorescence staining

Immunofluorescence staining was performed as previously described [54], but with some optimization. Briefly, coronal sections (4 μm) were baked in an oven for 2 h at 68 °C, deparaffinized in xylene, rehydrated *via* alcohol gradients, and washed with deionized water. Antigen retrieval was then conducted and sections were blocked in 5% bovine serum albumin for 45 min at room temperature. After blocking, sections were incubated with primary antibodies as follows: goat anti-Iba1 (1:300; Ab5076, Abcam), mouse anti-NeuN (1:400, MAB377, Millipore), rabbit anti-Tim-3 (1:100, ab185703, Abcam), rabbit anti-Nrf2 (1:200, ab89443, Abcam), or rabbit anti-HMGB1 (1:100, 3935S, Cell Signaling Technology) overnight at 4 °C. After washing with PBS (0.1 M, pH 7.4), sections were incubated with secondary antibodies: donkey anti-mouse Alexa 488 (1:400; A32766, Invitrogen), donkey anti-rabbit Alexa 555 (1:400; A31572, Invitrogen), donkey anti-mouse Alexa 555 (1:400; A32773, Invitrogen), donkey anti-goat Alexa555 (1:400; A21432, Invitrogen), or donkey anti-rabbit Alexa 488 (1:400; A21206, Invitrogen) for 1 h at room temperature. After washing with PBS three times, sections were incubated with 4’ 6-diamidino-2-phenylindole for 15 min at room temperature to stain nuclei. Finally, sections were viewed and images were captured under a fluorescence microscope (Leica-DMI8, Leica Microsystems, Wetzlar, Germany).

### Immunohistochemical staining

Immunohistochemical staining was performed as described previously [55]. Briefly, coronal sections (4 μm thickness) were deparaffinized, rehydrated, and soaked in a 3% hydrogen peroxide (H_2_O_2_) solution for 10 min at room temperature to quench endogenous peroxidase activity. Next, sections were blocked in 5% bovine serum albumin for 30 min and were incubated overnight at 4 °C with the following primary antibodies: rabbit anti-Tim-3 (1:100, ab185703, Abcam), rabbit anti-Nrf2 (1:200, ab89443, Abcam), or rabbit anti-HMGB1(1:100, 3935S, Cell Signaling Technology). The next day, the sections were washed with PBS three times for 10 min each and were incubated with biotinylated goat anti-rabbit IgG (1:100, ZSGB-Bio, Beijing, China) for 30 min at room temperature. Sections were then incubated with horseradish peroxidase streptavidin for 10 min and developed with diphenylamine peroxidase substrate. Finally, sections were viewed and images were captured under a light microscope (Leica-DM2500, Leica Microsystems).

### Terminal deoxynucleotidyl transferase-mediated biotinylated-dUTP nick-end labeling (TUNEL) staining

TUNEL staining was used to investigate cell apoptosis as previously reported. TUNEL staining was performed 24 h after SAH, as previously described with minor modifications [56]. Briefly, the cells surrounding the contused brain tissue were identified using an *In Situ* Cell Death Detection Kit (Roche, Nutley, NJ, USA) according to the manufacturer’s instructions. The slices were viewed and images were captured under a fluorescence microscope (Leica-DMI8, Leica Microsystems).

### Enzyme-linked immunabsorbant assay (ELISA)

At 24 h post-SAH, rats were anesthetized and transcardially perfused with 250 mL pre-cooled PBS (0.1 M, pH 7.4). Brains were then removed and rapidly divided into the left and right hemisphere; the left hemisphere was frozen in an ultra-low temperature refrigerator, homogenized in PBS (0.1 M, pH 7.4) at 200 mg/mL, and centrifuged at 12,000 g for 15 min. The liquid supernatant was collected and stored at −80 °C until further use. The concentrations of TNF-α, IL-1β, IL-17and IL-18 were analyzed using specific ELISA kits (BMS625, BMS630, and BMS629, respectively, Invitrogen) according to the manufacturer’s instructions. The concentration of cytokines was expressed by the intensity of absorbance measured by spectrometry on a microplate reader (Varioskan Lux, Thermo Scientific). Results were expressed in picograms per milliliter (pg/ml).

### RT-qPCR

At 24 h post-SAH, rats were deeply were anesthetized and transcardially perfused with 250 mL pre-cooled PBS (0.1 M, pH 7.4). Brains were removed and divided into left and right hemispheres; the left brain hemisphere was frozen at −80 °C for until further use. Approximately 100 mg of brain tissue was homogenized in 1 ml Trizol reagent and RNA extraction was performed as previously described. The concentration of total RNA was detected by an ultraviolet spectrophotometer and then reverse transcribed to cDNA using a PrimeScript RT reagent kit with gDNA Eraser (RR047A, Takara Bio Inc., Shiga, Japan), according to the manufacturer’s instructions. For RT-qPCR, 10 uL of total reaction, 1 uL of cDNA, 0.8 uL of primers, 3.2 uL of dH_2_O, and 5 uL of SYBR Premix Ex TaqTMII (RR820A, Takara Bio Inc.) were mixed and run on an Applied Biosystems 7500 Real-time PCR system. Program steps: pre-denaturation, 95 °C, 30 s; extension 95 °C, 5 s; and annealing 68 °C, 30 s, for 40 cycles. The mean gene expression level was quantified with standard samples and normalized to β-actin. The primer sequences were as follows:

for rat *Tim*-3,

sense primer: GGGCTAAGATCGGTAGGTGC,

antisense primer: CCATCTAGGCTAAGGTGGCG

for rat *GAPDH*,

sense primer: AGTGCCAGCCTCGTCTCATA

antisense primer: GATGGTGATGGGTTTCCCGT

### Statistical analysis

All data are represented as mean ± standard deviation (SD) and were analyzed using SPSS version 19.0 software (SPSS, IBM, Armonk, NY, USA). Mann-Whitney U or Student’s t tests were used to check differences between two groups after checking for normal distribution. One-way analysis of variance (ANOVA) with post-hoc Tukey’s test or Kruskal-Wallis followed by Dunnett’s test were used for assessing multiple comparisons. P < 0.05 was considered statistically significant. Cartograms were drawn using GraphPad Prism 5 software (GraphPad Software, Inc, San Diego, CA, USA).

### Ethics approval and consent to participate

All experimental procedures and animal care were approved by the Southern Medical University Ethics Committee and were conducted in accordance with the policy of the National Institutes of Health on the care and use of animals.

### Availability of data and materials

The datasets used and/or analyzed during the current study are available from the corresponding author on reasonable request.

## Supplementary Material

Supplementary Tables

Additional File 1
