# Supplementary material for: Tim-3 deteriorates neuroinflammatory and neurocyte apoptosis after subarachnoid hemorrhage through the Nrf2/HMGB1 signaling pathway in rats
Source: Aging (Albany NY). 2020 Nov 7;12(21):21161–85. doi: 10.18632/aging.103796 (PMC7695377; doi:10.18632/aging.103796)
Supplement: Supplementary Tables [file aging-12-103796-s001..pdf]

SUPPLEMENTARY TABLES

Supplementary Table 1. Study design and animal usage.

Parts	Groups	BBB	Brain Ebema	WB/ RT-qPCR	IHC/IF	Death	Mortality (SAH)	Exclude (SAH)	Sum
Exp.1	sham	-	-	6	3	-		-	
	SAH3h,6h,12h,24h,48h,72h	-	-	36	3	7		3	
Exp.2	sham	6	6	6	3	-		-	
	SAH	6	6	6	3	2		-	
	SAH+AAV-NC	6	6	6	6	3		2	
	SAH+AAV-Tim-3	6	6	6	6	9		1	
Exp.3	sham	6	6	6	3	-		-	
	SAH	6	6	6	3	-	11.19%	-	365
	SAH+siRNA-scramble	6	6	6	6	2		1	
	SAH+Tim-3-siRNA	6	6	6	6	1		-	
Exp.4	sham	6	6	6	3	-		-	
	SAH	6	6	6	3	1		-	
	SAH+AAV-Tim-3+DMSO	6	6	6	6	11		2	
	SAH+AAV-Tim-3+NK-252	6	6	6	6	2		-	
	Subtotal	72	72	114	60	38		9	

Supplementary Table 2. Mortality of different groups.

Groups	Rats used	Mortality
sham	72	（0）0
SAH	112	（10）8.92%
SAH+AAV-NC	27	（3）11.11%
SAH+AAV-Tim-3	33	（9）27.27%
SAH+siRNA-scramble	26	（2）7.69%
SAH+Tim-3-siRNA	25	（1）4.00%
SAH+AAV-Tim-3+DMSO	35	（11）16.12%
SAH+AAV-Tim-3+NK-252	26	（2）7.69%
