# Supplementary material for: Tim-3 deteriorates neuroinflammatory and neurocyte apoptosis after subarachnoid hemorrhage through the Nrf2/HMGB1 signaling pathway in rats
Source: Aging (Albany NY). 2020 Nov 7;12(21):21161–85. doi: 10.18632/aging.103796 (PMC7695377; doi:10.18632/aging.103796)

**Additional File 1.Grading System for SAH.**

| **Grade** | **Amount of subarachnoid blood** |
| --- | --- |
| 0 | no subarachnoid blood |
| 1 | minimal subarachnoid blood |
| 2 | moderate blood clot with recognizable arteries |
| 3 | blood clot obliterating all arteries within the segment |

| **Quadrant** | **Grade** |
| --- | --- |
| 1 |  |
| 2 |  |
| 3 |  |
| 4 |  |
| 5 |  |
| 6 |  |
| **Total:** |  |


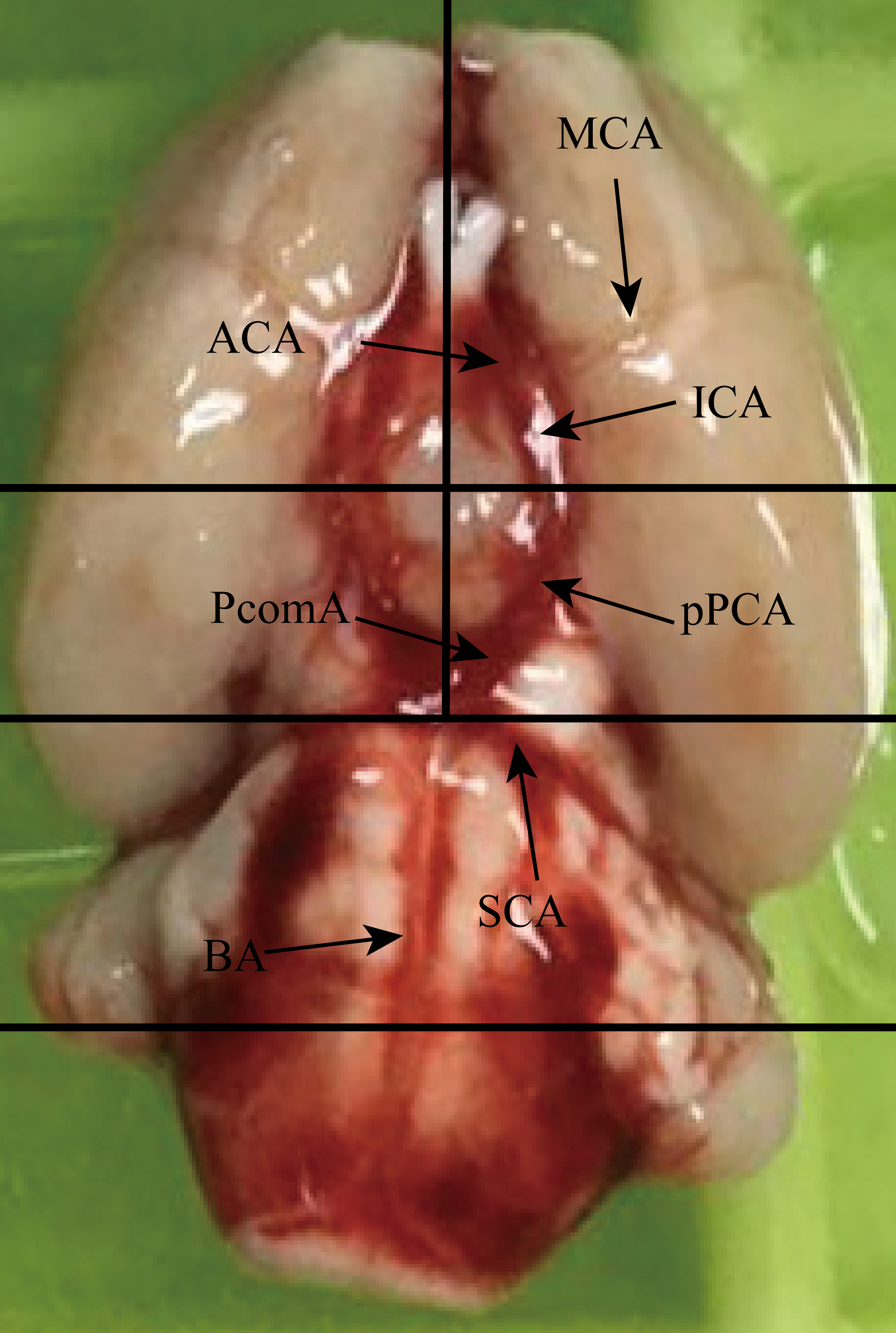

Supplement: Additional File 1 [file aging-12-103796-s002..docx]
